# Combined 3-dimensional printing model and 3-dimensional fluoroscopic navigation to assist C2 pedicle screw insertion: A case report

**DOI:** 10.1097/MD.0000000000021838

**Published:** 2020-10-23

**Authors:** Hao-Tian Xu, Shuang Zheng, Rong-Peng Dong, Tong Yu, Jian-Wu Zhao

**Affiliations:** Department of Spine Surgery, The Second Hospital of Jilin University, Changchun, Jilin Province, China.

**Keywords:** 3-dimensional, fluoroscopic, navigation, pedicle screw, rapid prototyping

## Abstract

**Rationale:**

The misplaced cervical screw can cause catastrophic surgical complications, such as nerve root damage, vertebral artery compromise, spinal cord injury, and even paraplegia. Thus, the present study aims to describe a novel technique of 3-dimensional printing model (3DPM) combined with 3-dimensional fluoroscopic navigation (3DFN) to facilitate C2 pedicle screw insertion.

**Patient concerns:**

A 56-year-old male patient presented hypoesthesia of the trunk and extremities, accompanied by a walking disorder.

**Diagnoses:**

Congenital atlantoaxial malformation with atlantoaxial dislocation.

**Interventions:**

He underwent an occipital cervical fusion. We used 3DPM and 3DFN technology to guide C2 pedicle screws insertion.

**Outcomes:**

We inserted 2 pedicle screws and 4 lateral mass screws using the combined 3DPM and 3DFN technology. All screws were classified as excellent position postoperatively. The surgical duration, total fluoroscopic time, and the bleeding volume were 258 minutes, 3.9 minutes, and 237 mL, respectively. No surgical complications, such as neurological compromise, nonunion, dysphagia, infection, polypnea, fixation failure, pseudarthrosis formation, or revision surgery, were observed. The follow-up duration lasted 30 months.

**Lessons:**

The combination of 3DPM and 3DFN to promote C2 pedicle screws implantation is a safe, accurate, reliable, and useful technology, which can achieve an excellent therapeutic effect and avoid surgical complications. However, using the 3DPM and 3DFN technology may increase the financial burden of patients.

## 1 Introduction

The cervical pedicle screw internal fixation system has the advantages of excellent biomechanical stability, stable internal fixation and high fusion rate.^[1–6]^ It was widely used for cervical diseases, such as cervical fracture, cervical dislocation, cervical disc herniation, cervical canal stenosis, cervical intraspinal tumors, cervical inflammatory diseases, and congenital cervical deformities.^[6–11]^

The traditional technique for pedicle screw insertion is predominantly based on the understanding of the cervical anatomical structure by the surgeon.^[4,5,12–14]^ Intraoperatively, the screw position can be determined by radiography. However, radiography lacks stereoscopic perception and real-time feedback. Besides, the acquisition of radiographs requires multiple adjustments of C-arm in anteroposterior and lateral positions, which may interrupt the surgical process and increase radiation exposure. Furthermore, the low resolution of the radiographic images makes it difficult for doctors to identify the insertion point of the needles. The authors reported that the freehand technique has a 14% to 23% screw malposition rate.^[4–6,12]^ Among them, 2.7% to 3.3% of patients experienced surgical complications, including vertebral artery compromise, nerve injury, and even mortality.^[6,15]^

Varieties techniques have been developed to promote pedicle screw insertion, including computer-aided surgery,^[1,16–18]^ guiding devices surgery,^[19]^ 3-dimensional rapid prototyping technology,^[20,21]^ robot-assisted operation,^[2]^ and augmented reality navigation surgery.^[3]^ However, these methods still have 1.8% to 11.4% screw malposition rate.^[19,20,22,23]^ Consequently, to maximize the safety of the operation, we combined 3-dimensional printing model (3DPM) and 3-dimensional fluoroscopic navigation (3DFN) to aid screws insertion in the cervical spine.

## 2 Ethics

This study was approved by the ethics committee of the Second Hospital of Jilin University, Changchun, China. The patient provided written informed consent for this report, and his information has been anonymized.

## 3 Case report

A 56-year-old male patient came to our clinical office who complained about hypoesthesia of the extremities and accompanied by a walking disorder. Besides, he denied any experience of cervical trauma.

### 3.1 Physical examination

We found that the patient has a claudication gait. The palpation of the neck revealed tenderness and percussion pain. Hypoesthesia of the skin was observed in the left upper arm, right hand, and bilateral lower extremities. The muscle strength of bilateral deltoid, bilateral biceps, bilateral triceps, and bilateral fissure was grade III. The power of bilateral iliopsoas muscle, bilateral quadriceps femoris, and bilateral tibial anterior muscle were all grade IV. Bilateral knee tendon reflexes and bilateral Achilles tendon reflexes are hyperactive. Both Hoffman signs are positive, and both Babinski signs are negative. Lasegue test and Bragard sign of bilateral lower extremities were negative.

### 3.2 Radiographic examination

**Figure 1:**
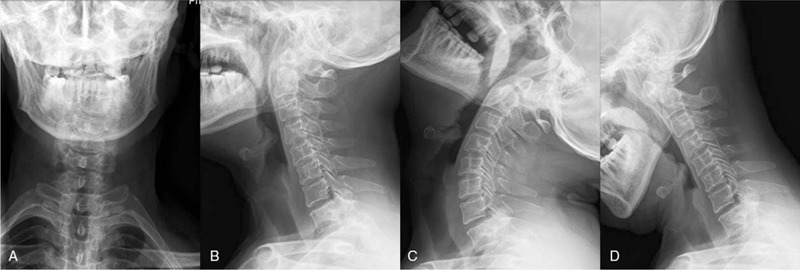
Cervical radiography plains. (A) anteroposterior and (B) lateral radiographs showed abnormal morphology of the C2 vertebral body; (C, D) dynamic radiographs revealed poor stability of C1-C2 segments.

**Figure 2:**
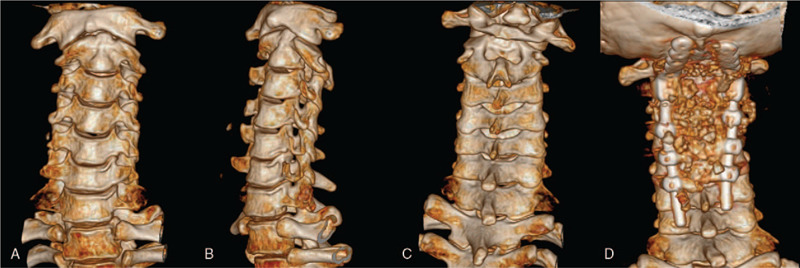
Three-dimensional CT reconstruction. (A–C) Preoperative 3-dimensional CT showed a slight anterior lateral dislocation of the atlas and an unstable atlantoaxial position. (D) Postoperative 3-dimensional CT. CT = computer tomography.

**Figure 3:**
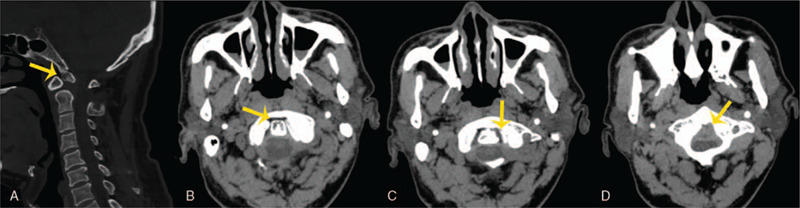
Three-dimensional CT. (A) The base and apex of the odontoid process were separated; (B–D) the left and right atlantoaxial space are narrow and partially fused.

**Figure 4:**
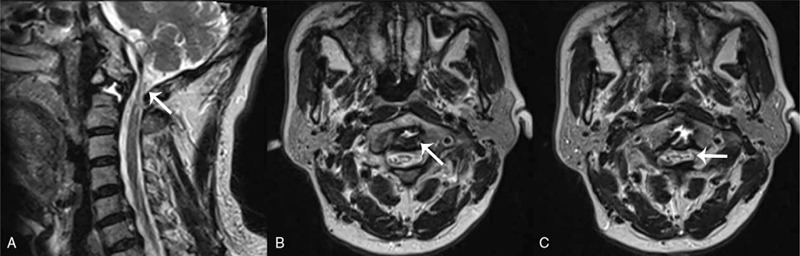
The T2-weighted phase of MRI. The sagittal image showed compression of the spinal cord at the C1-C2 segment and strip-like high signal in the spinal cord (A), and the axial images revealed severe cervical stenosis at the C1-C2 segment (B, C). MRI = magnetic resonance imaging.

Radiograph plains displayed a slight anterior dislocation of the atlas and an unstable atlantoaxial (Fig. 1A–D). Computed tomography (CT) arteriography showed no stenosis or filling defects in bilateral vertebral arteries, bilateral internal carotid arteries, bilateral common carotid arteries, and basilar arteries. Three dimensional CT was reconstructed (Fig. 2A–D) and demonstrated that the base and apex of the odontoid process was separated, the left and right atlantoaxial space was narrow (Fig. 3A–D). The magnetic resonance imaging images showed compression and strip-like high signal in the spinal cord (Fig. 4A) and severe cervical stenosis (Fig. 4B and C) at the C1-C2 segment. Thus, the patient was diagnosed as congenital atlantoaxial malformation, atlantoaxial dislocation, and secondary cervical stenosis.

### 3.3 Surgical technique

**Figure 5:**
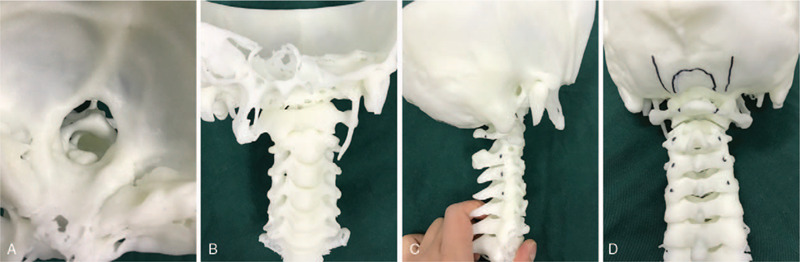
3D rapid prototyping. (A–D) the C1 and C2 vertebrates were observed on the 3D printed model from different angles, and the screws entry point and decompression range were designed before the operation. 3D = 3-dimensional.

Cervical spinal was scanned using CT (PHILIPS, the Netherlands), and the image data was recorded on the Compact Disc Read-Only Memory (CD-ROM) (Shenzhen Xinjinghua Technology Co., Ltd, Guangdong, China). We imported the image data in CD-ROM into the 3D printer for 3D modeling (Fig. 5A–D). Consequently, we could observe the anatomical structures of the spinal lesion authentically, dimensionally and adequately. Besides, we inserted CD-ROM into the workstation of 3D fluoroscopy navigation to design the trajectory, diameter, and length of the screws preoperatively.

**Figure 6:**
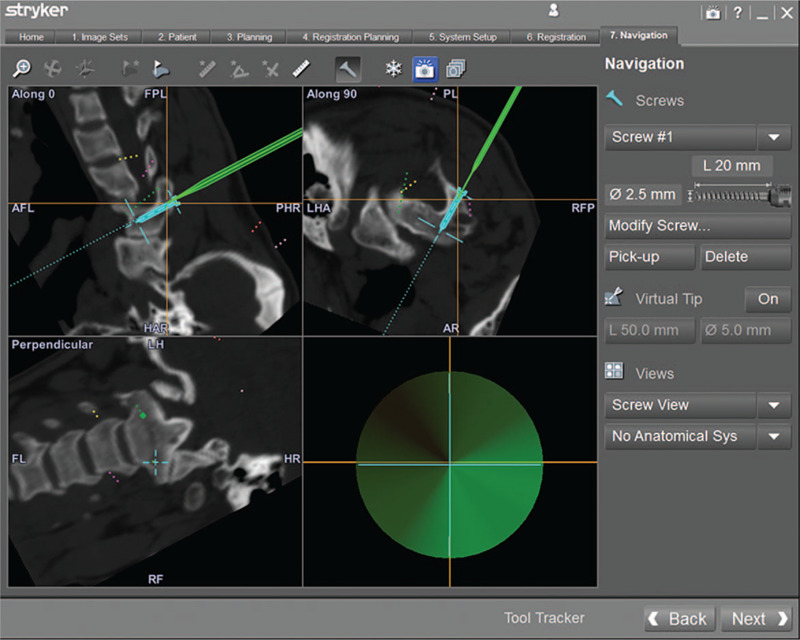
Intraoperative navigation image. The C2 pedicle screws were inserted with the assistance of 3DFN intraoperatively. 3DFN = 3D fluoroscopic navigation.

**Figure 7:**
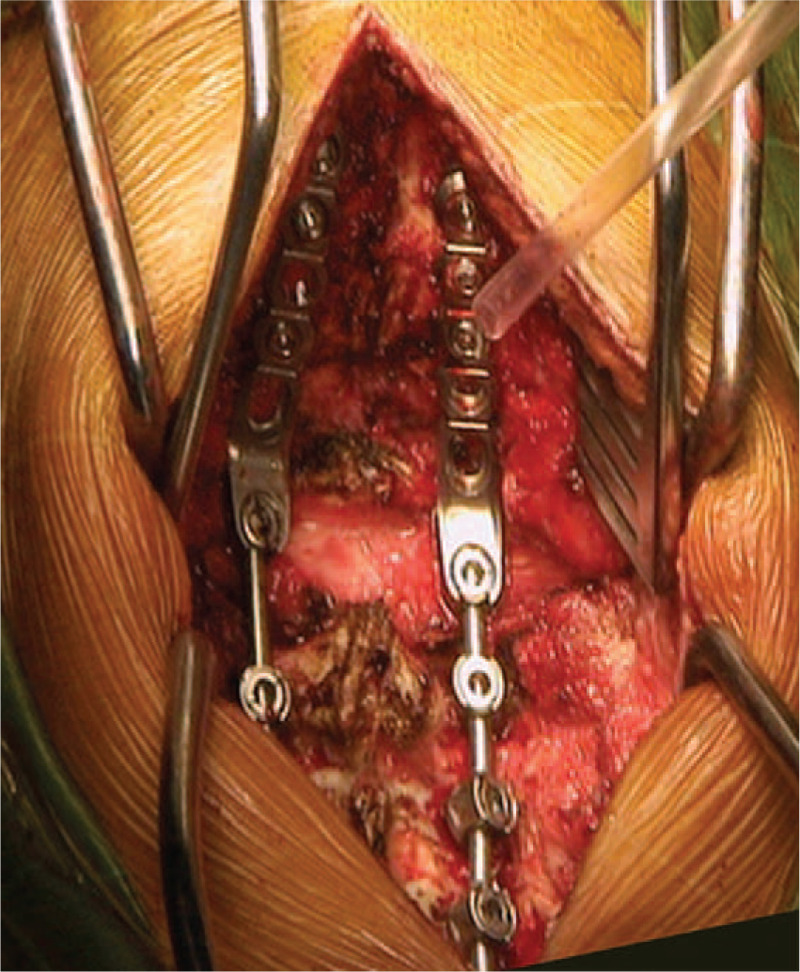
Intraoperative clinical pictures. The posterior atlas arch was removed to relieve spinal cord compression.

**Figure 8:**
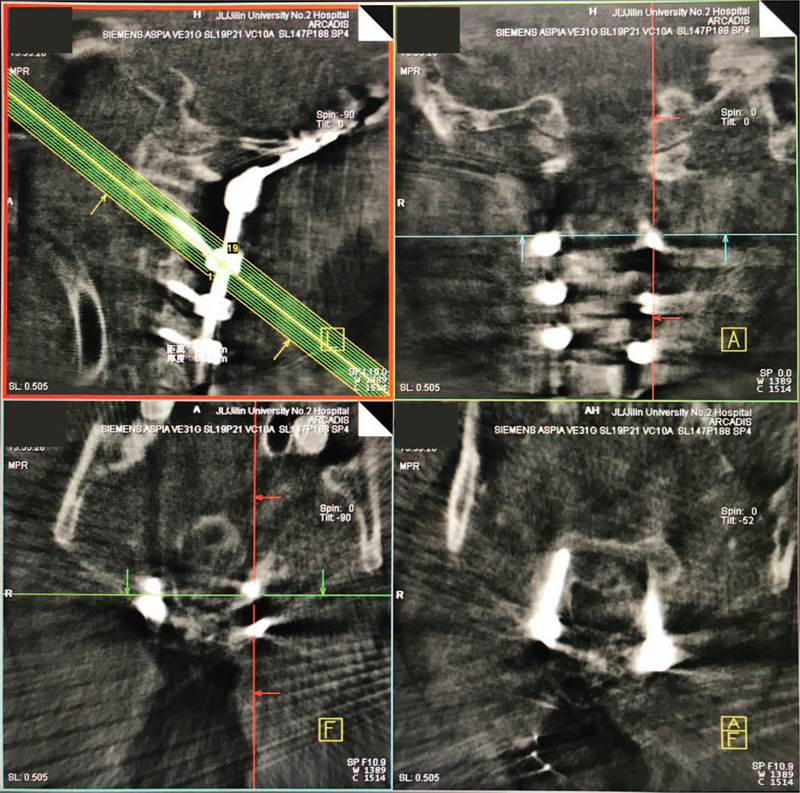
Intraoperative 3-dimensional fluoroscopy. The position of the fixation screws was evaluated using a 3D fluoroscopy of C-arm. C2 pedicle screws were graded as excellent. 3D = 3-dimensional.

After general anesthesia took effect, the patient was placed on a carbon fiber surgical table in a prone position. We selected the SpineMap 3D 2.0 software (Stryker Navigation, Kalamazoo, MI) in the navigation system. The surgeon attached the patient tracker (Stryker Leibinger GmbH & Co., Freiburg, Germany) to the C5 spinous process, then activated all navigation tools, including the patient tracker, C-arm tracker, and surgical tool tracker. Subsequently, the center of the C1-C2 segment was scanned 190° using the C-arm. Finally, we selected the screw view model of navigation to guide the screw insertion. The navigation monitor will indicate green when the direction of the surgical tool is the same as the preoperative design; it is the optimal trajectory to insert the C2 pedicle screws (Fig. 6). Besides, the surgeon can also observe 3DPM to improve their understanding of spinal lesions, to increase the accuracy of screws insertion, and to determine the range of decompression (Fig. 7). After all the fixation screws were inserted, the position of the fixation screws was evaluated by 3D fluoroscopy of C-arm (Fig. 8).

**Figure 9:**
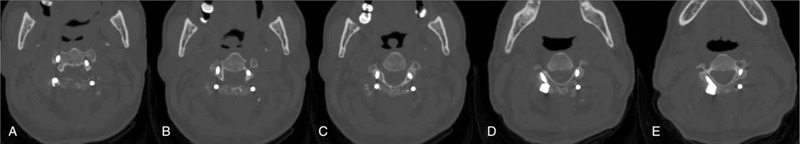
Postoperatively CT images. The (A–C) C2 pedicle screws, (D) C3, and (E) C4 lateral mass screws were all in an excellent position. CT = computer tomography.

We recorded the surgical period, the screw insertion time, the volume of blood loss, and the surgical complications. Besides, we estimated the position of the screws using CT scans (Fig. 9) postoperatively that described by Ughwanogho E et al.^[24]^ The patient was discharged 5 days after surgery.

### 3.4 Clinical outcomes and follow-up

The follow-up visit lasted 30 months postoperatively. Six screws were inserted and presented an excellent position, including 2 pedicle screws in C2, 4 lateral mass screws in C3 and C4. The surgical duration, total fluoroscopic time, and the bleeding volume were 258 minutes, 3.9 minutes, and 237 mL, respectively. No surgical complications, such as neurological compromise, nonunion, dysphagia, infection, polypnea, fixation failure, pseudarthrosis formation, or revision surgery, were observed.

## 4 Discussion

The pedicle screws and lateral mass screws are widely used in cervical spinal surgery.^[1,4–6,11,12,25,26]^ However, cervical pedicle is very narrow, which increases the difficulty of screw insertion. Moreover, the cervical pedicle is also adjacent to the spinal cord, vertebral artery, nerve root, and other vital structures, which makes the screw placement greatly hindered. Thus, the pedicle screw or lateral mass screw misplacement can cause damage to the spinal cord, dura, vertebral arteries, and nerve roots.

To increase the accuracy of screw insertion, computer navigation-assisted surgery, and 3DPM are used in spinal surgery.^[1,2,6,18,20,21,27,28]^ Sakai Y et al^[22]^ described that the navigation-assisted surgery is more accurate than freehand pedicle screw insertion, but it still has an 11.4% incidence of pedicle puncture. Yang et al^[20]^ suggested that the 3DPM can help surgeons to observe the anatomical structure of the lesion and simulate the operation before surgery; however, there is still a rate of 16.9% pedicle penetration. Therefore, we combined the benefits of 3DPM and 3DFN to improve the safety of pedicle screws insertion.

Concerning the accuracy of screws placement, Costa et al^[26]^ used 3D imaging-based navigation to assist screws insertion for the treatment of C1–2 traumatic fractures and found a 7.4% malposition rate. In our study, all the 6 screws in this study were in excellent positions, which was more accurate than the previous studies.^[4,5,12,14,15,17,25,26]^ We believe that this is partly related to the relatively low number of cases in this study, and also associated with the application of 3DPM combined with 3DFN technology. We suggest that the combination of 3DPM and 3DFN technology can effectively promote screw implantation and prevent neurovascular injury.

Regarding the operation time, Yang et al^[29]^ and Li et al^[25]^ believe that both 3DPM and computer navigation-assisted surgery can shorten the operation time. However, Harel et al^[25]^ compared the screws implantation time of C1-C2 guided by O-arm navigation (5 patients) and fluoroscopy (9 patients) and found that the latter has a shorter surgical duration (128 minutes vs 101 minutes). In the current study, the operation time was 258 minutes, which consistent with the previous literature reported by Jing et al^[18]^ and Zhao et al.^[1]^ We attribute this optimistic clinical outcome to a full understanding of the anatomical structures via the 3DPM and successfully determine the trajectory of screws by 3DFN.

As for blood loss, the amount of blood loss is influenced by the duration of operation,^[25]^ vascular injury,^[6]^ and intraoperative blood pressure. Hitti et al^[27]^ reported that navigation surgery could significantly diminish blood loss when compared to non-navigated surgery. The common reason for the vertebral artery injury is screw misplacement. Thus, accuracy screw placement is critical. Besides, surgeons should minimize intraoperative bleeding as allogeneic blood transfusions may bring the risk of infection and hemodynamic complications.^[1]^ In the present study, to minimize blood loss and shorten operation time, we applied lateral mass screws fixation in C3 and C4 segment. The amount of blood loss was 237 mL without any arterial injury, and we believe that the use of 3DPM and 3DFN technology is beneficial to reduce blood loss.

Screws loosening is a common surgical complication of spinal surgery with an incidence of 7% to 19.5%.^[15,30]^ Various risk factors are associated with screws loosening, including neck trauma, screw breakage, and pseudarthrosis formation postoperatively. We found no screws loosening in this patient during 30 months follow up visit. We attribute this excellent result to the optimal placement of the screws assisted by 3DPM and 3DFN. Besides, we prescribed a neck brace for the patient within 3 months after surgery.

Although this study has achieved good clinical outcomes, it still has some shortcomings. Most importantly, the combination of 3DPM and 3DFN can seriously increase the financial burden of patients. A large-sample multicenter randomized controlled study should be conducted to evaluate the clinical value of this technology. Besides, we performed 2 times of 3-dimensional C-arm scan during the operation, but we did not record the radiation exposure of the patient. Therefore, it is worthwhile to evaluate the radiation dose of the patient further.

The combination of 3DPM and 3DFN to promote C2 pedicle screws implantation is a safe, accurate, reliable, and effective method. It can achieve an excellent therapeutic effect and avoid surgical complications. However, combined 3DPM with 3DFN technology can increase the financial burden of patients.

## Acknowledgments

The authors gratefully acknowledge the cooperation of the doctors and nurses in the operating room.

## Author contributions

**Conceptualization:** Hao-Tian Xu, Tong Yu.

**Methodology:** Hao-Tian Xu, Shuang Zheng, Rong-Peng Dong.

**Software:** Shuang Zheng.

**Supervision:** Jian-Wu Zhao.

**Validation:** Rong-Peng Dong.

**Writing – original draft:** Hao-Tian Xu, Shuang Zheng, Tong Yu.

**Writing – review & editing:** Jian-Wu Zhao.
